# Reviewer acknowledgement 2015

**DOI:** 10.1186/s40697-016-0097-6

**Published:** 2016-02-17

**Authors:** Adeera Levin, Catherine M. Clase, Elizabeth Dicks, Manish M. Sood

**Affiliations:** Department of Medicine, Faculty of Medicine, The University of British Columbia, Vancouver, BC Canada; Department of Medicine, Faculty of Medicine, McMaster University, London, ON Canada; Memorial University of Newfoundland, St. John’s, NL Canada; Ottawa Hospital Research Institute, University of Ottawa, Ottawa, ON Canada

## Abstract

The Editors of *Canadian Journal of Kidney Health and Disease* would like to thank all our reviewers who have contributed to the journal in Volume 2 (2015).

**Mohsen Agharazii**

Canada

**R Todd Alexander**

Canada

**David Allan**

Canada

**Sunil Badve**

Australia

**Sean Bagshaw**

Canada

**Cheryl Barnabe**

Canada

**Lianne Barnieh**

Canada

**Marissa Battistella**

Canada

**Robert Allan Bear**

Canada

**Lorraine Bell**

Canada

**Vladimir Belostotsky**

Canada

**Geoffrey Block**

USA

**Guillaume Bollée**

Canada

**Branko Braam**

Canada

**Dylan Burger**

Canada

**Heloise Cardinal**

Canada

**Zoccali Carmine**

Italy

**Christopher Chan**

Canada

**Edward Clark**

Canada

**Anne-Marie Coté**

Canada

**Simon Davies**

UK

**Maryam Demian**

Canada

**Andre Denault**

Canada

**Meghan Elliott**

Canada

**Jean Ethier**

Canada

**Janine Farragher**

Canada

**Marie-Chantal Fortin**

Canada

**Bethany Foster**

Canada

**Amit Garg**

Canada

**Steven Greenway**

Canada

**Silviu Grisaru**

Canada

**Hind Harrak**

Canada

**Sunny Hartwig**

Canada

**Swapnil Hiremath**

Canada

**Rachel Holden**

Canada

**Steve Holt**

Australia

**Kristine Hommel**

Denmark

**Yoshitaka Isaka**

Japan

**Meg Jardine**

Australia

**Kati Kaartinen**

Finland

**Bhavneet Kahlon**

Canada

**Bryce Kiberd**

Canada

**Paul Komenda**

Canada

**Ngan Lam**

Canada

**Francis Lau**

Canada

**Louis-Philippe Laurin**

Canada

**John Lieske**

USA

**Jennifer MacRae**

Canada

**Dana Miskulin**

USA

**Gordon Moe**

Canada

**Amber Molnar**

Canada

**Catherine Morgan**

Canada

**Annie-Claire Nadeau-Frédette**

Canada

**Marta Novak**

Canada

**Ann O'Hare**

USA

**Georges Ouellet**

Canada

**Lars Penne**

Netherlands

**Maury Pinsk**

Canada

**Carol Pollock**

Australia

**Cassianne Robinson-Cohen**

USA

**Adam Romanovsky**

Canada

**Caren Rose**

Canada

**Guy Rousseau**

Canada

**Ruth Sapir-Pichhadze**

Canada

**Kara Schick-Makaroff**

Canada

**Samuel Silver**

Canada

**Sunita Singh**

Canada

**Amy Sood**

Canada

**Rosalie Starzomski**

Canada

**Rita Suri**

Canada

**Maarten W Taal**

UK

**Navdeep Tangri**

Canada

**James Tee**

USA

**Ben Thomson**

Canada

**Anne Tsampalieros**

Canada

**Delphine Tuot**

USA

**Michel Vallée**

Canada

**Lori Wazny**

Canada

**Steven Weisbord**

USA

**Christine White**

Canada

**Darren Yuen**

Canada

**Jeffrey Zaltzman**

Canada

**Michael Zappitelli**

Canada

